# Serological Antibodies against Kidney, Liver, and Spleen Membrane Antigens as Potential Biomarkers in Patients with Immune Disorders

**DOI:** 10.3390/ijms25042025

**Published:** 2024-02-07

**Authors:** Leidi Hernandez-Suarez, Eguzkiñe Diez-Martin, June Egiguren-Ortiz, Roberto Fernandez, Aitor Etxebarria, Egoitz Astigarraga, Cristina Miguelez, Andoni Ramirez-Garcia, Gabriel Barreda-Gómez

**Affiliations:** 1 Department of Research and Development, IMG Pharma Biotech S.L., 48170 Zamudio, Spain; leidi@imgpharma.com (L.H.-S.); eguz@imgpharma.com (E.D.-M.); june@imgpharma.com (J.E.-O.); r.fernandez@imgpharma.com (R.F.); aitor.etxebarria@imgpharma.com (A.E.); egoitz.astigarraga@imgpharma.com (E.A.); 2 Department of Immunology, Microbiology and Parasitology, Faculty of Science and Technology, University of the Basque Country (UPV/EHU), 48940 Leioa, Spain; andoni.ramirez@ehu.eus; 3 Department of Pharmacology, Faculty of Medicine and Nursing, University of the Basque Country (UPV/EHU), 48940 Leioa, Spain; cristina.miguelez@ehu.eus; 4 Neurodegenerative Diseases Group, BioBizkaia Health Research Institute, 48940 Barakaldo, Spain

**Keywords:** inflammation, autoimmune disorders, reactive antibodies, microarray

## Abstract

Immune disorders arise from complex genetic and environmental factors, which lead to dysregulation at the cellular and inflammatory levels and cause tissue damage. Recent research highlights the crucial role of reactive antibodies in autoimmune diseases and graft rejection, but their complex determination poses challenges for clinical use. Therefore, our study aimed to ascertain whether the presence of reactive antibodies against membrane antigens in tissues from both animal models and humans could serve as biomarkers in patients with autoimmune disorders. To address this issue, we examined the binding profile of serological antibodies against a diverse panel of cell membranes from the spleen, liver, and kidney tissues of monkeys, rats, and humans. After developing the cell membrane microarrays, human sera were immunologically assayed. The study was first conducted on sera from two groups, healthy subjects and patients with inflammatory and autoimmune disorders, and then optimized for kidney transplant patient sera. A significant increase in antibody reactivity against specific monkey kidney and spleen membranes was observed in the serum of patients with lupus nephritis, while kidney transplant patients showed a significant enhancement against human tissues and human embryonic kidney 293 cells. These results show the potential importance for clinical and basic research purposes of studying the presence of specific IgG against membrane antigens in patients’ serum as potential biomarkers of immune disorders. However, it is important to note that these results need to be verified in further studies with a larger sample size to confirm their relevance.

## 1. Introduction

Inflammation constitutes a crucial element among the defense mechanisms of the human body, being the process by which the immune system detects and eliminates foreign and harmful stimuli [1,2,3]. Inflammation can manifest as either an acute or a chronic response [4]. On the one hand, acute inflammation is a response of the innate immune system mainly due to external agents such as microorganisms, injuries, trauma, or toxic agents, and constitutes one of the first mechanisms of defense [5,6]. It is composed of cellular components like macrophages, neutrophils, dendritic cells, and natural killer cells [5,6,7], many of which migrate to the areas where the antigen or injury is found and initiate the innate immune response [8]. Additionally, inflammation includes blood proteins such as complement and blood coagulation systems to help in the development of the process and in the destruction of the external agent [7,9]. In contrast, chronic inflammation is not only a consequence of the innate response, but it is also largely mediated by the adaptive immune response that occurs due to the presence of both foreign and self-antigens [1,10,11]. The adaptive immune system is mainly composed of lymphocytes, which circulate in the blood and accumulate in secondary lymphoid organs, like lymph nodes and spleen, among others [12,13,14]. Chronic inflammation is the primary cause of most chronic diseases, including autoimmune disorders or inflammatory disorders, and poses a substantial threat to both health and longevity [1,15].

Autoimmune disorders are complex diseases characterized by deregulation at both the cellular and inflammatory mediator levels [16]. Certain genetic and environmental factors play a critical role in their development. In these conditions, the immune system mistakenly targets healthy cells due to dysfunction in the adaptive immune system [17,18,19]. In this context, self-reactive antibodies emerge as potential key indicators of autoimmune disorders, encompassing both autoantibodies and antibodies directed against external antigens [20,21,22]. The former primarily results from a genetic predisposition and is produced by B cells that possess the capability to identify and assail the internal components of the body [22,23]. Conversely, the latter arises due to the impact of environmental factors, like exposure to toxins, viruses, bacteria, and other infectious agents [23,24]. Conditions within this category include lupus nephritis (LN), systemic lupus erythematosus (SLE), ulcerative colitis (UC), Sjögren’s syndrome (SjS), rheumatoid arthritis (RA), and graft rejection as in kidney transplant (KT) patients, among others. These disorders share common characteristics such as an elevated degree of individual incapacitation, heightened immune activation, and persistent inflammation—either systemic or localized in specific organs—resulting in the impact on diverse tissues throughout the body [6,25].

Sex and age are two key factors that have been linked to the development of reactive antibodies in certain autoimmune diseases. Many autoimmune conditions like SLE, SjS, and type 1 diabetes exhibit a higher prevalence in females compared to males [26,27,28,29]. On the contrary, it has been observed that men with SLE more frequently suffer from renal impairments such as LN and face a heightened risk of progressing to end-stage renal disease, although there is some uncertainty surrounding this question [27,30,31]. The age at which first symptoms appear often dictates a less favorable prognosis in many of these diseases (SLE, type 1 diabetes) [32]. Early awareness of symptoms can aid healthcare professionals in delivering more effective treatment, potentially leading to improved outcomes.

The identification of self-reactive antibodies has proven to be valuable for both diagnosis and monitoring, as it facilitates the differentiation of autoimmune disorders, predicts disease progression, and anticipates potential complications [33]. This latter significance is underscored by the fact that the appearance of these antibodies in patients’ serum occurs long before these complications manifest [5,34]. Despite the constant need for more research in this field, the use of detection tools for these antibodies greatly improves the management of these disorders [20]. Enzyme-linked immunosorbent assays (ELISA) and antigen microarrays are generally used for their detection [33,35]. However, the purification of these antigens can modify their structure and, thus, their capacity to be detected by certain self-reactive antibodies, limiting their diagnostic potential. To overcome this limitation, the use of whole cells or membrane fractions can be a potential alternative, as membrane antigens are presented together with the other characteristic compounds of those membranes, such as lipids. The printing of whole cells or membrane fractions on glass slides has been used to study membrane proteins and lipids by radioligand binding studies, enzymatic analyses, or immunoassays [36,37,38], which have demonstrated that not only the structure and functionality of membrane proteins but also their antigenic profile seem to be preserved in these cell membrane microarrays (CMMAs).

Thus, this research aimed to ascertain whether the presence of reactive antibodies against membrane antigens in tissues from both animal models and humans could serve as biomarkers in patients with autoimmune disorders, particularly those with concurrent renal involvement. CMMAs were constructed, incorporating kidney, spleen, and liver tissues from rats, monkeys, and humans. The reactivity of sera from patients with autoimmune disorders against the antigenic panel of printed membranes was initially examined. Different autoimmune disease sera were incorporated to conduct a comprehensive sweep and ascertain whether the detected specific reactive antibodies were significantly relevant across various autoimmune diseases or, conversely, if they exhibited specific relevance only in certain conditions. Subsequently, only sera from renal transplant patients were analyzed, given the significance of reactive antibodies in graft rejection. In both cases, an optimization process was conducted to define the sample panel included in the microarrays and the immunological protocol employed. A significant binding of immunoglobulin G (IgG) to immobilized samples on the microarrays was achieved, demonstrating the effectiveness of the test in detecting these reactive antibodies. While these results underscore the promising potential of the technology, additional enhancements and future studies, including an expanded serum collection, are essential to confirm these findings. This validation is crucial for the eventual development of a diagnostic device that can aid physicians in the early detection and monitoring of patients with these autoimmune diseases.

## 2. Results

### 2.1. Detection of Self-Reactive Antibodies in Sera from Patients with LN and Other Autoimmune Disorders

A total of 28 sera were examined in the first part of the study. Of them, 12 came from healthy individuals used as a control group, 5 from patients who underwent kidney transplantation, and the remaining samples from individuals with the following autoimmune conditions: lupus nephritis, systemic lupus erythematosus, ulcerative colitis, Sjögren’s syndrome, rheumatoid arthritis, and chronic kidney disease (CKD) (Table 1). In the group of inflammatory disorders, samples from patients who underwent kidney transplantation were included, with the aim of observing whether there was an immune response that could indicate a potential rejection of the graft. To ensure the reproducibility and consistency of the results, each sample was analyzed using two replicates.

#### 2.1.1. Examination of the Responsiveness of Sera Categorized by Groups

**Figure 1:**
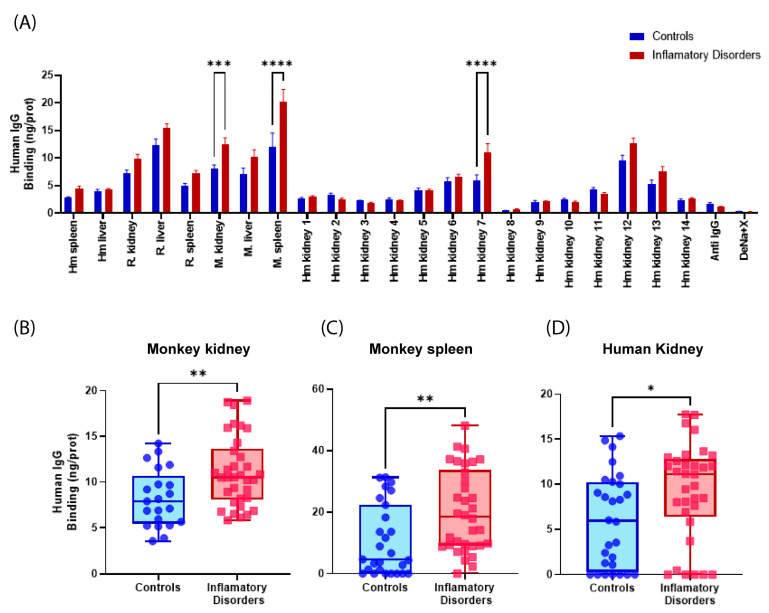
Binding of human IgG (ng IgG/pg prot) from both healthy individuals and patients with inflammatory disorders to various tissues. (**A**) Differences between healthy patients and patients with autoimmune disorders in all tissues; (**B**) in monkey kidney; (**C**) in monkey spleen; and (**D**) in human kidney. Rat (R), monkey (M), human (Hm). *p* < 0.05 (*); *p* < 0.01 (**); *p* < 0.001 (***); and *p* < 0.0001 (****).

Initially, the data was analyzed by dividing it into two groups. The first group encompassed all patients with inflammatory or autoimmune disorders. The second group comprised control patients. The normality of the samples was assessed using the Shapiro-Wilk test, setting significance at α = 0.05. The results indicated that the groups deviated from a Gaussian distribution. The binding of IgGs from the different serum groups to tissues immobilized on the CMMA was compared using a two-way ANOVA with Sídác’s post hoc multiple comparison test, α = 0.05. Significant differences (*p* < 0.05) were observed between the control group and the group of patients with autoimmune disorders in monkey kidney (mean ± SEM: 7.98 ng IgG/prot ± 0.75) and spleen tissues (4.89 ng IgG/prot ± 0.48), as well as human kidney tissue (7) (5.95 ng IgG/prot ± 0.98) (Figure 1A). Additionally, a *t*-test for unpaired samples was conducted to compare the serum groups, specifically in the tissues where significant differences were obtained with previously made two-way ANOVA, which revealed distinctions between both groups (Figure 1B–D).

#### 2.1.2. Reactivity of Each Serum to the Panel of Samples Immobilized in CMMAs—Heat Map

**Figure 2:**
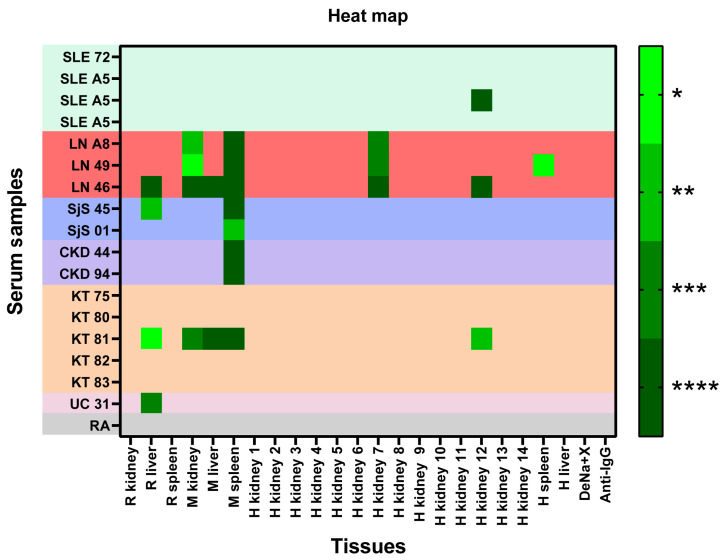
The heat map obtained from ordinary two-way ANOVA with Bonferroni’s multiple comparison test. SLE: systemic lupus erythematosus; LN: lupus nephritis; SjS: Sjögren’s syndrome; CKD: chronic kidney disease; KT: Kidney transplant; UC: ulcerative colitis; RA: rheumatoid arthritis; R: rat; M: monkey; H: human. *P* valor of this test is represented on the heat map: *p* < 0.05 (*); *p* < 0.01 (**); *p* < 0.001 (***); and *p* < 0.0001 (****).

To evaluate the reactivity of the 16 sera from patients with autoimmune disorders in each tissue, a two-way ANOVA with Bonferroni’s posthoc multiple comparison tests, α = 0.05, was carried out. The binding of IgG from the studied sera to the immobilized membrane panel on the CMMAs was higher in LN, SjS, and CKD. However, some sera from patients with KT, UC, and SLE exhibited certain reactivity. No signal was detected in the DeNa+X printing solution used as a blank. A heat map was generated using the *p*-values derived from the two-way ANOVA (Figure 2).

**Figure 3:**
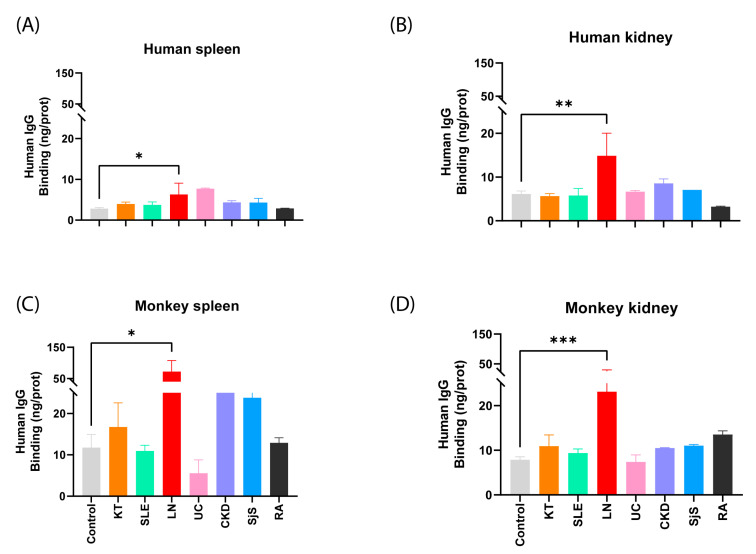
Binding of human IgG (ng IgG/pg prot) from both healthy individuals and patients with autoimmune disorders to various tissues. (**A**) Differences between healthy patients and patients with autoimmune disorders in the human spleen (ordinary one-way ANOVA with Holm-Sidák multiple comparison test); (**B**) in human kidney (ordinary one-way ANOVA with Dunnett’s multiple comparisons test); (**C**) in monkey spleen (Kruskal-Wallis test with Dunn’s multiple comparison test); and (**D**) in monkey kidney (ordinary one-way ANOVA with Dunnett’s multiple comparison test). *p* < 0.05 (*); *p* < 0.01 (**); and *p* < 0.001 (***). Control (grey); KT: kidney transplant (orange), SLE: systemic lupus erythematosus (green); LN: lupus nephritis (red); UC: ulcerative colitis (pink); CKD: chronic kidney disease (purple); SjS: Sjögren’s syndrome (blue); RA: rheumatoid arthritis (black).

In addition, the patients’ sera were examined by categorizing them according to their pathology. Then, the reactivity of these sera was analyzed and compared with the control group, specifically in the tissues with the highest binding of reactive antibodies. The normality of the samples was assessed using the Shapiro-Wilk test, with α = 0.05. A one-way ANOVA was conducted with Dunnett’s and Holm-Sidák post hoc multiple comparison test for sera that exhibited a normal distribution, while a Kruskal-Wallis test with Dunn’s post hoc multiple comparison test was employed for those with a non-parametrical distribution. The results obtained show significant differences for LN compared to the control group (Figure 3). The graph shows a high SD for the LN sera, which is due to the small size of the sample and the high reactivity of one of the sera.

#### 2.1.3. Evaluation of the Test Performance in the Detection of Reactive Antibodies

**Figure 4:**
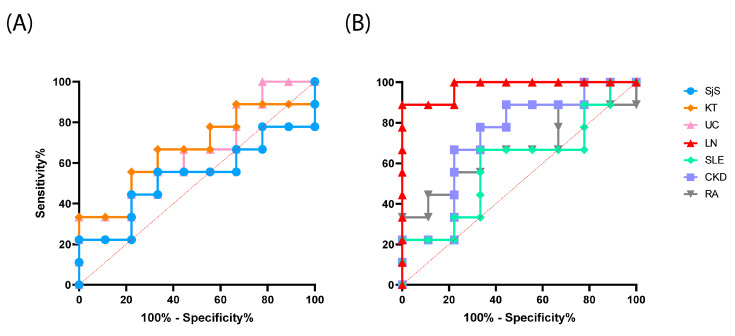
ROC analysis: (**A**) for autoimmune disorders (SjS, AUC = 0.5309 and CI 95%: 0.2479 to 0.8138; UC, AUC = 0.6543 and CI 95%: 0.3958 to 0.9128; and KT, AUC = 0.6667 and CI 95%: 0.4063 to 0.9270), and (**B**) for lupus nephritic (LN, AUC = 0.9753 and CI 95%: 0.9136 to 1.000) and for other autoimmune disorders (SLE, AUC = 0.5926 and CI 95%: 0.3188 to 0.8664; CDK, AUC = 0.7284 and CI 95%: 0.4843 to 0.9725; and RA, AUC = 0.6667 and CI 95%: 0.4043 to 0.9290). SLE: systemic lupus erythematosus; LN: lupus nephritis; SjS: Sjögren’s syndrome; CKD: chronic kidney disease; UC: ulcerative colitis; RA: rheumatoid arthritis; KT: kidney transplant. The dot lines indicate the reference diagonal, a line that connects the point (0,0) to (1,1) in the ROC plot and represents a scenario where the model’s predictive ability is no better than random chance.

Finally, an assessment of the test performance for reactive antibody detection in LN and other autoimmune disorders was conducted through a receiver operating characteristic (ROC) curve analysis [39,40]. The analysis revealed an area under the curve (AUC) of 0.98 specifically for LN compared to the control group, revealing notable sensitivity and specificity. However, the same high level of sensitivity and specificity was not achieved for the remaining studied autoimmune disorders (Figure 4).

### 2.2. Detection of Self-Reactive Antibodies in Sera from Patients with KT

A second part of the study was developed to evaluate the reactivity of sera from patients who received kidney transplants against a specific antigenic panel. This last one was composed of several human and rat spleen, liver, and kidney tissues, as well as monkey kidney tissue. In addition, HEK 293 cells were included. 

#### 2.2.1. Examination of the Responsiveness of Sera Categorized by Groups—PCA

**Figure 5:**
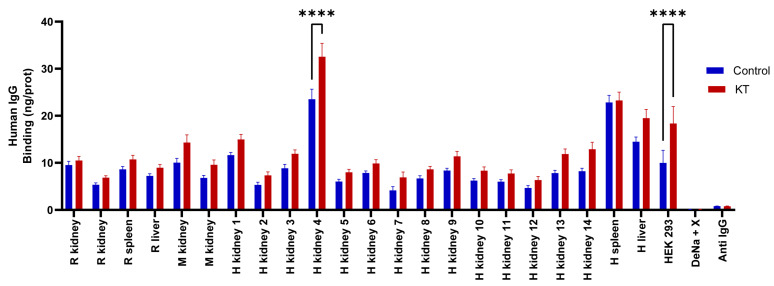
Binding of human IgG (ng IgG/pg prot) from both healthy individuals and patients with KT to kidney, liver, or spleen samples from rat (R), monkey (M), and human (H) (ng/prot). *p* < 0.0001 (****).

For the analysis, the sera were separated into two groups. The first group comprised all healthy patients, serving as control (*n* = 6). The second group consisted of patients with kidney transplants (*n* = 10) (Table 1). Each serum was analyzed in triplicate under identical conditions to validate the obtained results. The results obtained deviated from a Gaussian distribution according to the Shapiro-Wilk test; α = 0.05. The binding of IgGs from the different serum groups to tissues immobilized on the CMMA was compared using a two-way ANOVA with Sídác’s post hoc multiple comparison test, α = 0.05. Significant differences *(p* < 0.05) were observed between the control group and the group of patients with KT in human kidney tissue (4) and HEK 293 cells (Figure 5).

To assess the distinctions among all the tissues immobilized on the CMMAs in detecting reactive sera, a principal component analysis (PCA) was conducted. For the analysis, the top 10 variables that best described the variances among the samples were selected. PC4 and PC7 were chosen as they exhibited the highest classification accuracy among the components.

**Figure 6:**
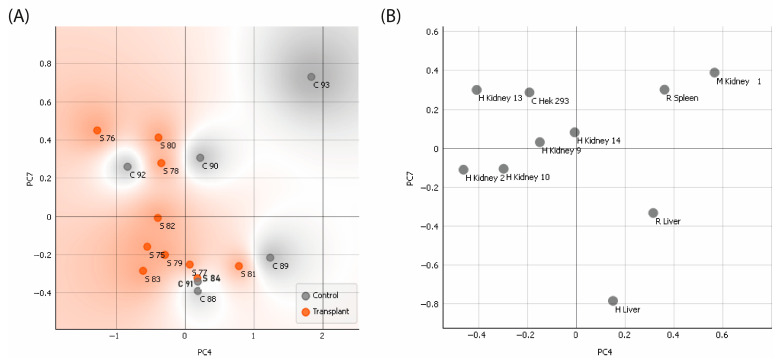
Principal component analysis (PCA) to examine the fourth and the seventh principal components (PC4, PC7) of the tissues. (**A**) Representing sera; (**B**) Representing tissues.

On the Figure 6A, all sera were depicted, including both control or negative sera in grey and transplanted or positive sera in orange. The graph illustrates the test’s ability to differentiate between different groups, identifying transplant sera and control sera as belonging to their respective groups. On the Figure 6B, the graph represents the ability of each human tissue to discriminate between positive and negative sera (Figure 6).

#### 2.2.2. Reactivity of Renal Transplant Patients’ Sera to the CMMAs Antigenic Panel

**Figure 7:**
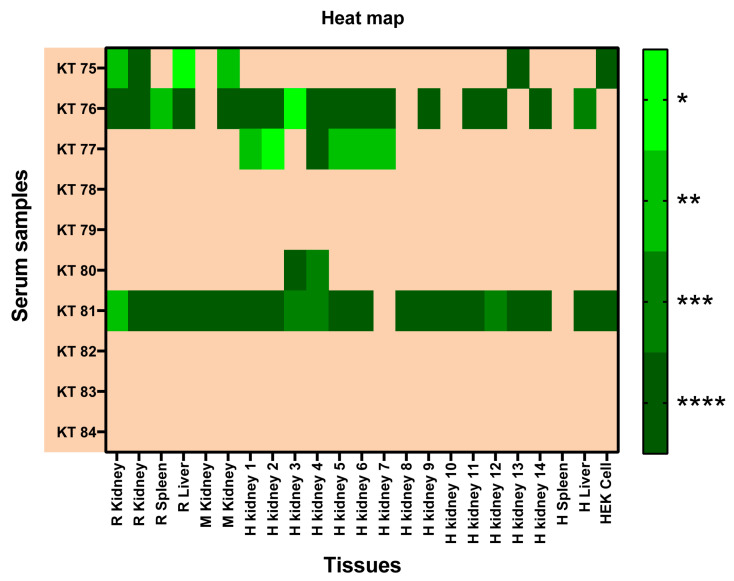
The heat map obtained from ordinary two-way ANOVA with Bonferroni’s multiple comparison test. The *p*-value of this test is represented on the heat map. KT: kidney transplant; R: rat; M: monkey; H: human. *p* < 0.05 (*); *p* < 0.01 (**); *p* < 0.001 (***); and *p* < 0.0001 (****).

To convey the reactivity of each serum in comparison to the control group against the antigenic panel sensor, a two-way ANOVA with Bonferroni’s post hoc multiple comparison test, α = 0.05, was carried out. The serum of patients 76 and 81 displayed the greatest reactivity against the panel of antigens included in the CMMAs. Sera 75, 77, 78, 79, 80, 82, and 83 also exhibited some reactivity as well but only in specific samples. In this context, HEK 293 cells and human kidney tissues showed the highest binding of reactive IgG from the sera analyzed. No signal was detected in the DeNa+X printing solution used as a blank. A heat map was generated using the *p*-values derived from the two-way ANOVA (Figure 7).

#### 2.2.3. Evaluation of the Test Performance—Receiver Operating Characteristic Curve

**Figure 8:**
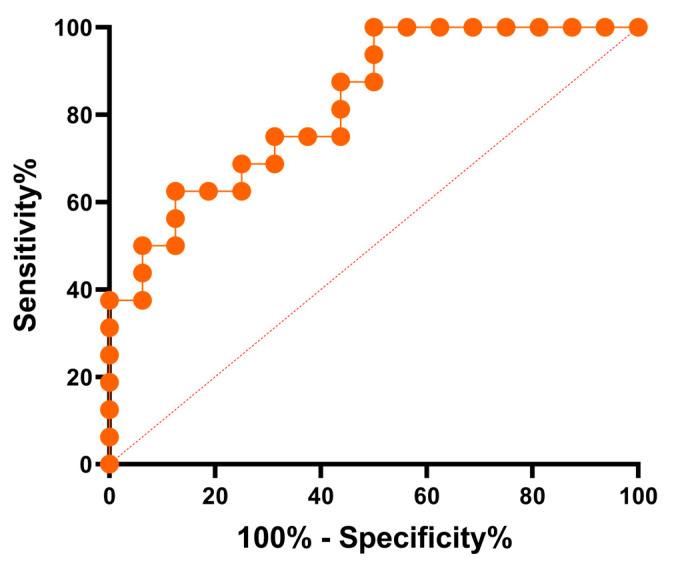
ROC curve analysis for the sera of KT patients vs. healthy controls (AUC = 0.82, and CI 95%: 0.6835 to 0.9650). The dot lines indicate the reference diagonal, a line that connects the point (0,0) to (1,1) in the ROC plot and represents a scenario where the model’s predictive ability is no better than random chance.

Using the acquired results, a ROC curve was conducted to assess the accuracy, sensitivity, and specificity of the test [39,40]. The analysis unveiled an area under the curve of 0.82, underscoring the remarkable sensitivity and specificity of the test in accurately identifying sera from patients who indeed possess reactive antibodies, as well as its capability to effectively exclude those who do not have these antibodies (Figure 8).

### 2.3. Examining the Influence of Sex and Age on Reactive Antibody Occurrence

**Figure 9:**
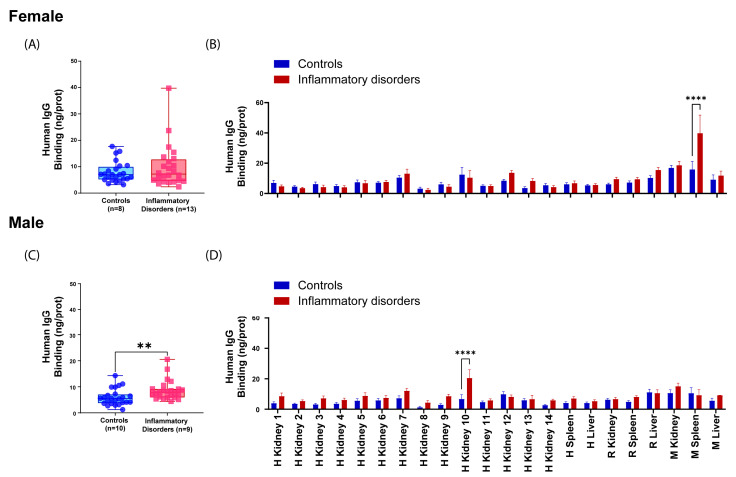
Binding of human IgG (ng IgG/pg prot) from both healthy individuals and patients with inflammatory disorders in females and males. (**A**) Differences between groups in females, *t*-test; (**B**) Differences between groups in all tissues in females, two-way ANOVA with Sídác’s post hoc multiple comparison test; (**C**) Differences between groups in males, *t*-test; (**D**) Differences groups in all tissues in males, two-way ANOVA with Sídác’s post hoc multiple comparison test; Rat (R), monkey (M), human (H). *p* < 0.01 (**); and *p* < 0.0001 (****).

The influence of sex and age on the occurrence of reactive antibodies was ultimately investigated. The aspect of age was explored in the study, and no statistically significant differences were detected. Likewise, the influence of sex on the occurrence of reactive antibodies was studied. Initially, serum reactivity was analyzed by comparing the control group and the inflammatory disorders group in both females and males using a *t*-test for unpaired samples. The analysis revealed significant differences (*p* < 0.05) between controls and inflammatory disorders in males but not in females (Figure 9A,C). Secondly, the binding of IgGs from the different serum groups to tissues immobilized on the CMMA was compared using a two-way ANOVA with Sídác’s post hoc multiple comparison test, α = 0.05. Significant differences (*p* < 0.05) were observed between the control group and the inflammatory disorders group in monkey spleen in females and human kidney tissue in males (Figure 9B,D).

## 3. Discussion

Despite recent advances in science in general and immunology in particular, diagnosing and managing autoimmune disorders remains a major challenge. This is attributed to the necessity for multiple tests to arrive at a diagnosis [33]. Additionally, the process can become quite sluggish due to the symptoms and damage not commonly manifesting at the onset of the disease; they may gradually appear over time or occur intermittently [41]. In this context, a tool like CMMAs to identify reactive antibodies in the serum of patients with autoimmune disorders during the initial phases of the disease could pave the way for improved monitoring and control of such patients, even in asymptomatic periods [38].

The first part of the study aimed to assess the presence of self-reactive antibodies in the serum of patients with autoimmune disorders such as LN, comparing them with sera from healthy individuals. CMMAs with an immobilized panel of human and animal membrane samples were used as antigenic panels to test diverse sera.

Animal tissues were incorporated into the study to assess whether comparable outcomes could be achieved in both human and animal tissues. This was prompted by the considerable complexity and cost associated with obtaining human tissue compared to animal tissue. The challenge of acquiring human samples, coupled with the high cost, makes it more convenient to procure a substantial quantity of animal tissues, thereby enhancing the reproducibility of the results. 

The tissues that exhibited a higher antigenic recognition by the sera were the kidney and spleen tissues from both monkeys and humans. This may be, principally attributed that the LN specifically target antigens in the kidneys. Regarding the spleen, the explanation may lie in the fact that the spleen, being a secondary lymphoid organ where the blood is filtered from pathogens and abnormal cells, could enhance the interaction between antigen-presenting cells and lymphocytes in response to the antigens in the sera [42].

The results obtained revealed that the IgG binding levels of sera from patients with LN to the spleen and kidney tissues of monkeys were comparable to those obtained with the corresponding human tissues.

Although this study assessed various autoimmune disorders, it was in the sera of LN patients where a higher reactivity was observed against the antigenic panel. These results may stem from the pronounced heterogeneity of LN compared to other autoimmune disorders under scrutiny that primarily target specific tissues or glands, such as ulcerative colitis [43,44,45]. LN is characterized by a high production of autoantibodies, which lead to the formation of immune complexes that are subsequently deposited in the glomerulus, initiating an inflammatory reaction and causing kidney damage over time [46,47,48,49,50]. Consistent with this elevation of reactive antibodies, and considering that LN involves antibodies specifically targeting the kidney [51,52,53,54], an increased reactivity of LN sera was observed against tissue samples included in the CMMAs. Sera from individuals with SLE, a disease known for its high heterogeneity [55], and with SjS were also included in the study, but no significant results were detected. In line with this observation, SjS is considered a systemic autoimmune disorder with not very common complications in the kidneys; only 5% of the total reported cases have renal complications [56,57], although the low n of our study would demand confirmation in further studies. Similar patterns are observed in RA, where kidney complications are more prevalent than SjS; however, no significant differences were identified in the study in this regard [58]. It is essential to highlight that these findings should be strengthened by expanding the number of individuals under evaluation.

A ROC curve analysis was conducted to assess the validity of the CMMA test [39,40]. According to the findings, it was confirmed that sera from patients with autoimmune disorders indeed exhibit higher reactivity compared to sera from healthy individuals, as widely reported in the literature [59,60,61,62,63,64]. Therefore, these reactive antibodies could potentially serve as markers to identify outbreaks of such pathologies and adjust medication, although it is acknowledged that there may be the presence of reactive antibodies in healthy patients [41]. The high sensitivity and specificity of the ROC curve reaffirm the validity of CMMA as a monitoring tool. This, in turn, confirms that serological antibodies against membrane antigens in spleen and kidney tissues, both in monkeys and humans, can be used as possible biomarkers in patients with autoimmune disorders. However, it is important to note that these results should be reinforced with a larger number of samples.

In order to study more specifically the role of reactive antibodies in a pathology in which the immune system is clearly involved, such as renal transplantation [5], a second part of the study was carried out with sera from kidney transplant patients. Sera from immunosuppressed patients were employed to analyze whether the activation of the immune system, associated with transplant rejection, could be detected through CMMA technology. The aim was to observe an increase in IgG binding following modifications made in the immunological protocol, which should not be evident due to their immunosuppressive treatment. Additionally, since these patients did not exhibit rejection symptoms at the time of sample extraction, the early detection of elevated IgG binding to CMMAs could serve as a potential predictor for transplant rejection.

Therefore, this second study was focused on assessing the immunological reactivity of sera from kidney transplant patients against an antigenic panel consisting of both animal and human tissues. Additionally, HEK 293 cells were included as part of the antigenic sensor. The inclusion of these cells was based on studies reported in the literature validating their use in various areas of experimentation [65]. These areas encompass studies on protein expression and interaction, viral packaging, and even antibody detection [65,66]. Moreover, since these cell lines are more readily available from commercial suppliers compared to tissues from both humans and animals, their incorporation into the study would enhance result reproducibility.

Differences between the control group and the KT group were scrutinized in the antigenic panel of tissues immobilized on the CMMAs. The results demonstrated a significant binding of IgG from the sera of KT patients to HEK 293 cells and certain human kidney tissues. Although HEK 293 cell lines are used as kidney epithelial cell lines [66], they also express some proteins specific to immature neurons, e.g., neurofilament proteins or alpha-internexin [67]. Hence, it is still unclear whether they should be considered a model of kidney cells or whether they should be considered instead as immature neurons [65,67,68,69]. Given this information, the significant rise in IgG binding to this cell line could be attributed to both its expression of inflammatory biomarkers (neurofilament proteins [70]) and its representation of human kidney epithelium. Anyhow, it is suggested that HEK 293 cells could be a great tool to be utilized during the monitorization of KT patients, enhancing the prediction capability of clinicians to detect possible kidney transplant rejection. 

The sensitivity and specificity of the test to detect reactive antibodies in the tissue panels studied were determined. For that, a PCA and a ROC curve were carried out, validating the functionality of the CMMAs. Therefore, this demonstrates that the presence of specific IgG against membrane antigens in patients’ serum can be used as a possible biomarker of kidney transplant rejection. 

It is well known that reactive antibodies play a crucial role in graft survival in KT [71,72,73,74]. This is due to its direct relationship with rejection and, therefore, decreased long-term graft survival. For these reasons, it is vital to maintain monitoring of the recipient’s immune response to the graft [75].

Monitoring the immune response in transplant patients is a common practice during post-transplant treatment. The assessment includes the evaluation of transplant stability, renal function, and the recipient’s immune system sensitization to the new organ [76,77]. Additionally, managing immunosuppressive treatment is a crucial aspect to monitor due to its role in long-term graft survival. While reducing it could decrease toxicity and risks of opportunistic infections, it might elevate the risk of immunological rejection [5,78]. Therefore, achieving an appropriate balance is crucial. Currently, various methods exist for monitoring these patients, ranging from biopsies, serum creatinine levels, and immunoassays to even predictions based on artificial intelligence [77].

Biopsies, despite being the gold-standard technique for monitoring transplant survival, are highly invasive. On the other hand, monitoring of serum creatinine levels lacks sensitivity and specificity, although they are less invasive than biopsies. Additionally, once these levels of creatinine manifest, the damage to the graft may already be irreversible [77]. Hence, the detection of reactive antibodies against the graft through immunoassays is highly promising. ELISA-type techniques are the preferred ones, renowned for their high precision and sensitivity [33,79]. Nevertheless, their performance is overshadowed by the fact that their analysis times are lengthy, requiring qualified and experienced personnel as well as specialized laboratory equipment, and none are available with the antigenic profile used in the present study. Furthermore, ELISAs require a higher volume of reagents and samples, making it a less cost-effective technique than CMMA, given the type and origin of samples that are used as antigen fingerprints [33]. Consequently, having tools that overcome these drawbacks, like the CMMA technology, would enhance the prognosis for these patients and streamline the management of the disease and its treatment [38,80].

Moreover, considering the known impact of sex on the appearance of reactive antibodies [26,27,28,30], this study also evaluated this variable in the analyzed sera. The objective was to determine whether specific tissues offer more valuable information for male patients while others exhibit greater specificity for diagnosis in female patients. This analysis revealed a notable binding of IgG to monkey spleen membrane antigens in females, contrasting with a stronger binding of IgGs to the human kidney in males. Although this premature analysis necessitates further expansion for a more precise conclusion, the results suggest the potential for a personalized approach to testing based on the patient’s sex. In other words, the appearance of IgGs in certain tissues, along with the sex variable, could be used as an indicator [26,27,28,29,30]. Regarding age, no differences were found in this study. However, it is important to acknowledge that future studies designed to explore the effects of various variables may reveal additional insights beyond those presented here. It is worth noting that the design of this study was specifically tailored to assess the utility of the CMMA platform for studying IgG binding.

The use of CMMAs to monitor patients with inflammatory disorders proves advantageous, as demonstrated in this study. However, it is crucial to acknowledge the limitations of this research. One drawback is the limited number of samples studied, necessitating future studies with a more significant sample size to corroborate the obtained results and draw conclusive insights into the potential significance of specific IgG presence against membrane antigens in patient sera as possible biomarkers for immune disorders.

Aspects to improve in future studies include the exclusive fabrication of CMMA using only animal tissues and HEK 293 cells. This approach could enhance result reproducibility and address challenges associated with obtaining human tissues, as demonstrated in previous studies with CMMAs [38,80,81]. However, it is essential to consider that an optimal approach for monitoring and evaluating the immune system response would involve a test crafted with the patient’s own tissue. This personalized testing method holds the potential to provide more accurate insights into the individual’s immune system dynamics. Additionally, as highlighted earlier, conducting future studies with a larger sample size is crucial to confirm the results and pave the way for the development of Point-of-Care Tests (POCT) that would be beneficial for both patients and physicians in the future. 

In summary, this study underscores the significant utility of conducting the serological analysis of IgG binding to membrane antigens in patients with inflammatory disorders, although further studies are needed to overcome the mentioned limitations. Moreover, the CMMA tool has certain advantages over the reference techniques currently used, such as the large number of tests that can be produced with the sample extracted from a biopsy, which could pave the way for the development of a customized test using the patient’s own membranes. These tests would enable the monitoring of reactive antibodies against the affected target tissue over time, providing relevant information on the patient’s humoral response and its behavior in response to treatments, being especially useful in inflammatory processes such as transplantation.

## 4. Materials and Methods

### 4.1. Serum Samples

Throughout this study, a total of 39 human serum samples were utilized. Among them, 21 belonged to patients with inflammatory disorders (F: 12; M: 9), supplied by BioIVT (Frankfurt, Germany). Additionally, eighteen control sera were examined (F: 8; M: 10). Of these, 6 were provided by BioIVT (Frankfurt, Germany), and 12 originated from the Biobank of the Aragon Health System (Zaragoza, Spain). This study was evaluated by relevant ethics committees and obtained approval (Approval ID: C.I. PI23/291).

The samples were stored at −80 °C until analysis. The characteristics of these sera are shown in the following table (Table 1). It should be noted that KT patients were under immunosuppression treatment.

### 4.2. Tissue Samples

Tissues from human kidney, spleen, and liver biopsies were supplied by the AMSBIO (Abingdon, UK) tissue bank according to its ethical protocols (Table 2). The tissues were obtained from Caucasian patients, specifically from the healthy sections of the tissue. 

Likewise, kidney, liver, and spleen tissue samples from male monkeys of the species *Macaca fascicularis*, approximately 4 years old, from the CIMA (Applied Medical Research Center of the University of Navarra, Navarra, Spain) were included in the study. In addition, tissues of the kidney, spleen, and liver of rats were included as a control. They were supplied by the University of the Basque Country (Leioa, Spain). Lastly, HEK 293 cells that were used in the last part of the study were obtained from Sigma-Aldrich (Darmstadt, Germany).

### 4.3. Design and Methodology of the Study

This study aimed to assess the reactivity of sera from patients with autoimmune disorders, as well as from patients who underwent kidney transplantation. The study was divided into two parts. In the first part, the antigenic profile of sera from patients with LN and other autoimmune disorders was investigated. The second part focused on sera from renal transplant patients. These sera were assessed against an antigen panel comprising membranes from various animal and human tissues, presented in the form of CMMAs. The objective was to determine whether these reactive antibodies could serve as biomarkers to identify these pathologies. To achieve this, different configurations of CMMAs were designed and tailored to the specific requirements of each part of the study.

CMMAs were composed of a collection of cell membrane homogenates isolated from different tissues, including the kidney, liver, and spleen of rats, monkeys, and humans. The design incorporated two replicas of each mentioned tissue. Additionally, HEK cells were immobilized along with previous tissues for the second study. Furthermore, duplicate prints of various known concentrations of rat cerebral cortex were included to facilitate the quantification of protein in the printed samples. Additionally, different concentrations of human IgG were printed to calculate the amount of reactive IgG at each point. An Anti-Human IgG was also printed to indicate the total IgG content in each serum sample. Finally, DeNa+X, the printing solution employed in the process, was included as a blank. Animal tissues were integrated into the design of CMMAs in order to observe if comparable results could be achieved in both human and animal tissues. This approach was chosen due to the complexity and high cost associated with acquiring human tissue samples. The manufacturing process of microarrays involves two key steps. Initially, membrane homogenates are prepared, and upon completion of this step, the printing process is carried out. After the printing process and subsequent validation of the utility of the CMMAs, the reactivity of the sera was determined through an immunological assay. Subsequently, the obtained results were quantified and subjected to statistical analysis.

### 4.4. Cell Membrane Microarray Fabrication

Briefly, samples were homogenized using either a disperser (Ultra-Turrax^®^ T10 basic, IKA, Staufen, Germany) or a Teflon-glass grinder (Heidolph RZR 2020, Schwabach, Germany) in 20 volumes of homogenization buffer (1 mM EGTA, 3 mM MgCl_2_, and 50 mM Tris-HCl, pH 7.4) supplemented with 250 mM sucrose. The crude homogenate underwent a 3857× *g* centrifugation (AllegraTM X 22R centrifuge, Beckman Coulter, Brea, CA, USA) for 5 min at 4 °C. The collected supernatant was centrifuged at 18,000× *g* (Microfuge^®^ 22R centrifuge, Beckman Coulter, Brea, CA, USA) for 15 min at 4 °C. The pellet was washed in 20 volumes of homogenized buffer and subjected to a second centrifugation under the same conditions. Subsequently, the supernatants were finally discarded, and the pellets were frozen at −80 °C until the fabrication of CMMAs, except for one aliquot, which was reserved for determining the protein concentration. Protein concentration was determined by the Bradford method [82] and adjusted to the final concentration. 

Membrane homogenates were resuspended in a printing buffer and printed onto glass slides using a noncontact microarrayer (Nanoplotter NP 2.1, GESIM, Radeberg, Germany), depositing two replicates of each sample (30 nL/spot) onto pre-activated glass microscope slides [83]. Membrane homogenates of each tissue were obtained from different individuals. The printing process was carried out under controlled humidity (relative humidity 60%) at a controlled temperature of 4 °C. CMMAs were stored at −20 °C until usage [80]. Prior to usage, CMMAs underwent validation through various methods [84,85]. These acquired CMMAs were utilized to quantify the IgG levels present in the different sera through an immunoassay.

### 4.5. Immunoassay Procedure

**Figure 10:**
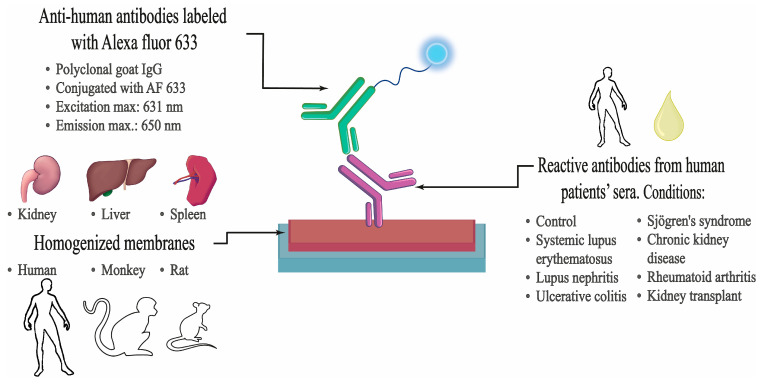
Immunoassay procedure. Information on the CMMA and the samples used.

For the first part of the study, the following protocol for detecting reactive antibodies in the sera of patients with autoimmune disorders was implemented. First, the slides were defrosted and dried for 30 min at room temperature in a drying chamber. Subsequently, fixation was performed with 70% methanol (Thermo Fisher Scientific, Waltham, MA, USA) at −20 °C for 10 min. Then, the slides were washed twice for 5 min with phosphate-buffered saline (sodium dihydrogen phosphate dehydrated 77 mM, sodium hydrogen phosphate monohydrated 24.2 mM, sodium chloride 1.4 M, pH 7.4) with 0.25% Triton (*v*/*v*) (0.25% PBS-T) on a slide envelope with shaking. The microarrays were then incubated with blocking solution (5% normal goat serum (NGS) (Vector Laboratories, New Ark, CA, USA), in 0.25% PBS-T) for 30 min at room temperature, followed by one-hour incubation with AffiniPure Fab Fragment Goat Anti-Human IgG (H+L) (Jackson ImmunoResearch, West Grove, PA, USA). Microarrays were incubated overnight at 4 °C in a slide humidity chamber with the sample diluted 1:200 in blocking solution. After incubation, the slides were washed three times with 0.25% PBS-T for 5 min each in a slide envelope with shaking. The slides were then dried with a fan for 10 min and incubated for 1 h with goat anti-human IgG Alexa fluor 633 secondary antibody (Life Technologies Corporation, Carlsbad, CA, USA) at 2 mg/mL in 2.5% NGS and 0.25% PBS-T, at room temperature in a dark humidity slide chamber. With this incubation, we guarantee that only the IgG of the sera bound to the immobilized tissue will be detected. After incubation, the slides were washed twice with 0.25% PBS-T and twice with 1× PBS for 5 min each in a slide envelope with shaking. Finally, dipping was carried out in distilled water. The slides were dried with a fan, and the fluorescent signal was revealed using a ChemiDoc: Universal Hood 3 imaging system (BioRad, Hercules, CA, USA) with Green EPI laser illumination and a 605/650 nm filter. The subsequent figure illustrates the sequence of steps that were undertaken (Figure 10).

In the second phase of the study, multiple optimizations were implemented, building upon the primary or preceding protocol. These optimizations encompassed modifications in incubation and washing times, along with adjustments to the working concentrations. First, the slides were defrosted and dried for 30 min at room temperature in a drying chamber. Subsequently, fixation was performed with 70% methanol at −20 °C for 10 min. Then, the slides were washed three times for 10 min with 0.25% PBS-T on a slide envelope with shaking. The microarrays were then incubated with blocking solution for one hour at room temperature, followed by a one-hour incubation with AffiniPure Fab Fragment Goat Anti-Human IgG (H+L). The slides were washed three times for 10 min with 0.25% PBS-T on a slide envelope with shaking. Microarrays were incubated for two hours in a slide humidity chamber with the sample diluted 1:30 in blocking solution. After incubation, the slides were washed three times with 0.25% PBS-T for 10 min each in a slide envelope with shaking. The slides were then dried with a fan for 10 min and incubated for 1 h with goat anti-human IgG Alexa fluor 633 secondary antibody at 2 mg/mL in 2.5% NGS and 0.25% PBS-T at room temperature in a dark humidity slide chamber. After incubation, the slides were washed three times with 0.25% PBS-T and twice with 1× PBS for 5 min each in a slide envelope with shaking. Finally, dipping was carried out in distilled water. The slides were dried with a fan, and the fluorescent signal was revealed using a ChemiDoc: Universal Hood 3 imaging system with Green EPI laser illumination and a 605/650 nm filter.

### 4.6. Data Processing and Normalization

The signal was quantified using ImageScanner software version 2.2 (IMG Pharma Biotech S.L., Zamudio, Spain). Data handling and analysis were carried out using Excel version 2108 (Microsoft Corporation, Albuquerque, NM, USA) and GraphPad software version 9.2.0 (GraphPad Software, Dotmatics Inc, San Diego, CA, USA). For the microarrays, the analysis data obtained were normalized to the amount of total protein and were expressed as means of independent data points ± S.E.M. (standard error of the mean). The normality of the data was tested using the Shapiro–Wilk statistical test with α = 0.05.

### 4.7. Statistical Analyses

For Gaussian distributed data, a statistical analysis was performed using GraphPad software by two-way ANOVA with Bonferroni’s post hoc multiple comparison test. To analyze non-parametric data, the Kruskal–Wallis test with Dunn’s multiple comparison test was performed, also using GraphPad software. Statistical differences were indicated by *p*-values < 0.05.

The analysis carried out to evaluate the validity of the CMMA was developed through an ROC study using the GraphPad software. This approach was used to identify the optimal threshold involved in calculating the distance of each cut-off point from the coordinates (0, 1) in the upper left corner of the ROC space. At this time, the sensitivity was 100%, and the specificity of 1 was 0%; therefore, within the data set, the threshold value on the ROC curve closest to this point was considered the most optimal [39,40].

A PCA has been performed by transforming the 10 most descriptive variables in order to obtain a smaller number of them, the Principal Components (PCs), able to explain most of the variance. For the performance of these analyses, the Orange data mining toolbox [86] was employed. Then, the pair of PCs with the higher classification accuracy was selected.

## 5. Conclusions

In conclusion, this study successfully demonstrated the efficacy of CMMAs in detecting serological antibodies against membrane antigens in human kidney and spleen tissues, as well as in monkey tissues and HEK 293 cells. However, confirmatory studies are imperative to validate this technology, strengthening the findings through analysis of larger sample size and confirming its role in identifying potential rejection events or acute outbreaks of immune disorders. As a result, physicians could respond promptly, adjusting or altering therapy according to the patient’s needs.

## 6. Patents

The authors use the patent entitled ‘Method for the surface treatment of solid substrates’ and identified by the patent number EP 2 048 534 A1. This patent has been used during the development of CMMAs.

## Figures and Tables

**Table 1:** Information on the different human control serum samples and human serum samples from patients with autoimmune conditions was used for the development of the assay.

Condition	Sex	*n*	Range Age
Lupus Nephritis	Female	3	32–45
Systematic Lupus Erythematosus	Female	2	51–55
Sjögren Syndrome	Female	2	54–55
Chronic Kidney Disease	Female	2	54
Rheumatoid Arthritis	Male	1	72
Ulcerative Colitis	Male	1	60
Kidney Transplant	Female	3	54–64
	Male	7	25–70
Control	Female	8	20–83
	Male	10	19–67

**Table 2:** Information on the different human tissue samples used for CMMA development.

Anatomical Site	Sex	*n*	Range Age
Liver	Male	1	46
Spleen	Male	1	48
Kidney	Male	8	38–64
	Female	6	46–70

## Data Availability

The data presented in this study are available on request from the corresponding author (privacy, legal and ethical reasons).
